# Synthesis, structural and morphological characterizations of nano-Ru-based perovskites/RGO composites

**DOI:** 10.1038/s41598-019-43726-1

**Published:** 2019-05-28

**Authors:** Ahmed Galal, Hagar K. Hassan, Nada F. Atta, Ali M. Abdel-Mageed, Timo Jacob

**Affiliations:** 1 Department of Chemistry, Faculty of Science, Cairo University, 12613 Giza, Egypt; 2 Institute of Surface Chemistry and Catalysis, Ulm University, 89069 Ulm, Germany; 3 Institute of Electrochemistry, Ulm University, 89081 Ulm, Germany; 4 Helmholtz-Institute-Ulm (HIU), Helmholtzstr. 11, 89081 Ulm, Germany; 5 Karlsruhe Institute of Technology (KIT), P.O. Box 3640, 76021 Karlsruhe, Germany

**Keywords:** Materials chemistry, Graphene, Nanoparticles

## Abstract

Highly-dispersed Ru-based perovskites supported on reduced graphene oxide (*A*-RG) nanocomposites are prepared using different *A-*metal salts (Sr(NO_3_)_2_, Ba(NO_3_)_2_ and Ca(NO_3_)_2_). The procedure is based on a redox reaction between the metal precursors and graphene oxide (GO) using two different routes of reaction initiation: through thermal heating or by microwave-assisted heating. The resulting nanocomposites do not require further calcination, making this method less energy-demanding. In addition, no additional chemical reagents are required for either the GO reduction or the metal precursor oxidation, leading to an overall simple and direct synthesis method. The structure and morphology of the as-prepared *A*-RG (non-calcined) nanocomposites are characterized using various structural analyses including XRD, XPS, SEM/EDX and HR-TEM. Changing metal *A* in the perovskite as well as the “activation method” resulted in significant structural and morphological changes of the formed composites. SrRuO_3_ and BaRuO_3_ in combination with RuO_2_ are obtained using a conventional combustion method, while SrRuO_3_ (~1 nm size) in combination with Ru nanoparticles are successfully prepared using microwave irradiation. For the first time, a microwave-assisted synthesis method (without calcination) was used to form crystalline nano-CaRuO_3_.

## Introduction

Owing to their high catalytic activity, high thermal stability and high specific capacity, Ru-based materials proved useful in electrocatalysis^1,2^ and energy storage applications (*e.g*., in Li-ion batteries^3^ and supercapacitors^4–8^). Various methods are used for the preparation of Ru and/or RuO_2_-based materials such as hydrothermal^9^, sol-gel^10^, chemical reduction or oxidation^11,12^ and precipitation methods^13^. However, this class of materials is rather sensitive to the preparation parameters, and consequently the resulting structure, morphology and catalytic properties varies depending on the adopted preparation approach.

One drawback of using Ru-based materials in different applications is related to their low porosity and high preparation cost. To reduce the costs for commercial applications, usually highly-dispersed Ru-based particles are deposited on less expensive supports such as high surface area carbon materials^14^. This helps decreasing the overall costs of the Ru-based electrode materials and increasing the specific surface area and porosity. Several reports discussed the preparation of Ru or RuO_2_ over carbon materials. For instance, Lou *et al*. synthesized Ru nanoparticles over activated carbon, derived from *Moringa Oleifera* fruit shells for supercapacitor applications^15^. Their method is based on the thermal reduction of Ru^3+^ ions at high temperature using ZnCl_2_ as an activating agent for carbon formation. Also for supercapacitors, He *et al*.^14^ prepared hydrous RuO_*x*_ over activated carbon black by a chemical impregnation technique. They found that the specific capacitance is greatly affected by the mass loading of RuO_*x*_ as well as the specific surface area. Moreover, Zheng *et al*.^16^ reported that the performance of RuO_2_ as supercapacitor is highly altered by the calcination temperature. Carbon nanotubes were also used as a support for RuO_2_ nanoparticles prepared by the reaction of Ru(VI) and Ru(VII)^17^. Recently, graphene has been widely used as a support for RuO_2_^4–8,14^ due to its high surface area and outstanding thermal and electronic characteristics^14,18^. This makes graphene an interesting choice for many applications such as catalysis^19^, electronics^20^, sensors and biosensors^4,14^ as well as supercapacitors^4–8^.

Restacking or aggregation of graphene sheets limits reaching their theoretical surface area and thus decreasing its electrochemical efficiency^14^. Therefore, metal or metal oxide nanoparticles have been used as spacers for graphene sheets to prevent graphene restacking and to tune its properties^14^. As mentioned before, Ru and/or RuO_2_ have been widely used with graphene sheets especially in electrocatalysis and supercapacitor applications to overcome the problems of graphene restacking. To further improve the properties of metal oxide-based graphene composites doping of the graphene sheets turned out to be an attractive route^21^. Other approaches for forming graphene composites included Cu-metallic organometallic frameworks/reduced graphene oxide for improved performance of supercapacitors and sensitive sensing of nitrite^22^. Supercapacitors performance can be enhanced by modifying reduced graphene oxide with molybdenum disulfide^23^ and nitrogen-doped reduced graphene oxide-MnO_2_ nanocomposite prepared by hydrothermal method^24^.

Another approach involves the formation of mixed oxides^13^ or perovskite^25^. Perovskites have the structure of *AB*O_3_, where *A* is usually the cation with larger atomic radius (a rare-earth metal or an alkali metal), and *B* is the cation with smaller atomic radius (usually a transition metal)^26^. They have versatility in both their chemical compositions and structure flexibility^27^, leading to interesting unique properties^28–31^ that make them suitable for various applications such as superconductors^32^, energy storage devices^33^, catalysis^34^ and electrochemical sensors^35^. For the preparation of these perovskites several methods have been reported, including wet–chemical methods^34,35^, hydrothermal synthesis^36–38^, thermal decomposition^39^, sol-gel methods^40^ and polymerizable complex methods^41^. Again, by choosing a particular preparation method and by tuning the synthesis parameters, perovskites with diverse physical or chemical properties could be prepared^42^.

In the present contribution we introduce a new strategy to prepare Ru-based perovskites/graphene nanocomposites by applying an energy-saving synthesis approach. The method is based on the simultaneous redox reaction of a Ru precursor and GO in a single step without the need of any reducing agent. For catalyzing the redox reaction, we employed a thermal (*i.e*. combustion) and microwave-assisted initiation. In the combustion initiation, the metal precursors are heated with the fuel (GO) on a hotplate. In the microwave-assisted initiation, microwave irradiation operated at  720 W is used to promote the reaction. The as-prepared materials are directly characterized without performing any calcination process. We studied the effects of varying the preparation routes and of changing the *A*-site elements on the resulting structures and morphologies of the formed composites. The second part of this work aimed at evaluating the performance of the prepared materials for supercapacitors application^43^.

## Experimental

### Preparation of graphene oxide

Graphene oxide is prepared by a modification of Hummer’s method, following the same procedure reported by Kovtyukhova *et al*.^44^. Briefly, 5 g of high purity graphite is cured with H_2_SO_4_, P_2_O_5_ and K_2_S_2_O_8_ to prepare the pre-oxidized graphite. This is followed by stirring 5 g of dried pre-oxidized graphite with 115 mL of concentrated H_2_SO_4_ in an ice bath for 10 minutes. After that, 15 g of KMnO_4_ is gradually added and stirred for two hours. The mixture acquired a bright yellow color after dilution with water and treatment with H_2_O_2_. The bright yellow suspension is filtered and washed with 1:10 (v/v) HCl-solution, and finally dried overnight in an oven at 80 °C.

### Preparation of *A*RuO_3_/RGO nanocomposites by combustion method

Ru-based materials supported on the reduced graphene oxide (*A*-RG) are prepared by mixing 0.2 g of graphene oxide (GO) with 0.33 mmol of RuCl_3_ and 0.33 mmol of metal salt (Sr(NO_3_)_2_, Ba(NO_3_)_2_ or CaCO_3_ treated with concentrated HNO_3_ in 20 mL distilled water to form Sr-RG-C, Ba-RG-C or Ca-RG-C, respectively. The resulting suspensions are ultra-sonicated for two hours until a homogeneous mixture of metal precursor is attained. An ammonia solution is used to adjust the pH of the suspensions to 8.0 ± 0.05. The mixture is then heated on a conventional hotplate at 200 °C in air; the mixture dehydrated and transformed into a viscous mass that is followed by its self-ignition. The resulting composites are left on the hotplate for a total of two hours, which is the total reaction time. After the ignition process (firing takes place), black powders are obtained, which is an evidence of the successful reduction of RGO. For comparison, Ru/RGO was prepared by the same method but in absence of Sr, Ba or Ca salts.

### Preparation of *A*RuO_3_/RGO nanocomposites microwave method

The same solutions are prepared (as those created with combustion methods) by mixing 0.2 g of GO with 0.33 mmol of RuCl_3_ and 0.33 mmol of metal salt (Sr(NO_3_)_2_, Ba (NO_3_)_2_ or CaCO_3_ treated with conc. HNO_3_ in 20 mL distilled water to form Sr-RG-M, Ba-RG-M or Ca-RG-M, respectively. The mixtures are then placed in a conventional microwave (720 Watts) using 30 s-cycles (the microwave irradiation is switched on and off for 20 s and 10 s, respectively) until the ignition process takes place; the total reaction time is 30 minutes. During the irradiation, the suspension becomes viscous with time and dehydrated. The resulting powder is ignited, and a strong firing takes place, which generates a black powder that indicates the successful reduction to RGO.

### Structural, spectral and surface analyses

All prepared materials are characterized using X-ray diffraction (XRD), X-ray photoelectron spectroscopy (XPS), field-emission scanning electron microscopy (FESEM) with energy dispersive analysis by X-ray (EDX) and high-resolution transmission electron microscopy (HR-TEM). XRDs are recorded with Panlytical X’Pert using Cu-*K*_*α*_ radiation (*λ* = 1.540 Ǻ). The surface morphology is analyzed by HR-TEM (Tecnai G20, FEI, Netherland, 200 kV, LaB6 Gun) and FESEM with EDX (JEOL JSM-6360LA and Philips XL30). TEM measurements were performed on Tecnai G20, FEI, instrument, Netherland, 200 kV, LaB6 Gun. The particles size was calculated using Image J software. At least 500 particles were evaluated and collected from several shots for the same sample. The percentage of the particles was plotted against their size to generate particle size distribution curve.

XPS measurements are performed with a Perkin Elmer 5300 XPS system with a non-monochromatized Mg-*K*_*α*_ X-ray source. Calibration is performed using the C-1*s* component (binding energy of 284.6 eV). An Mg-*K*_*α*_ X-ray is used with 300 W applied to the anode. For the XPS peak deconvolution, the XPS Peak 4.1 software is used, while Shirley background is employed to subtract the background.

## Results and Discussion

The reduced graphene oxide (RGO) supported Ru-based perovskite composites are successfully prepared in the absence of any reducing or stabilizing agents under controlled pH synthesis conditions using different metal salts (Sr(NO_3_)_2_, Ba(NO_3_)_2_ or CaCO_3_) and two initiation methods. Elaborate characterization of the resulting materials including chemical composition and morphological characterization is accomplished using a combination of analysis techniques such as XRD, XPS, SEM-EDX and TEM.

### Sr-RG nanocomposites

**Figure 1:**
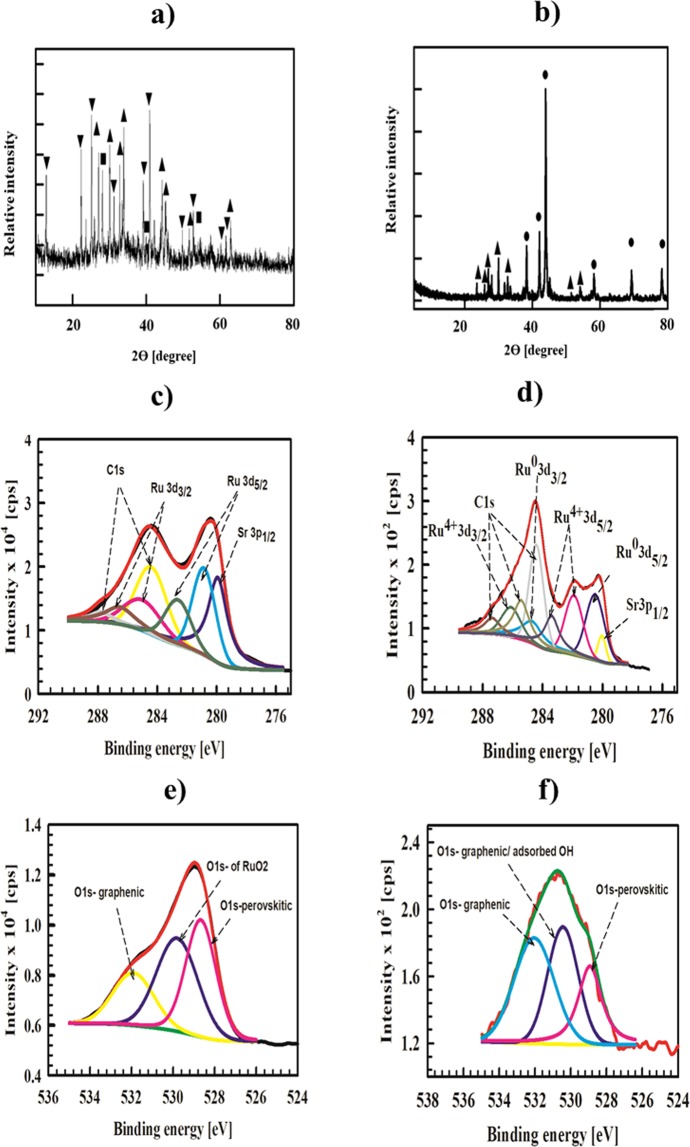
XRD of (**a**) Sr-RG-C, (**b**) Sr-RG-M, (▪ RuO_2_, ● metallic Ru, ▲ SrSO_4_ and ▼ SrCl_2_), (**c**) C 1*s*, Ru 3*d* and Sr 3*p* of Sr-RG-C, (**d**) C 1*s*, Ru 3*d* and Sr 3*p* of Sr-RG-M, (**e**) O 1*s* of Sr-RG-C and (**f**) O 1*s* of Sr-RG-M.

Figure (1a) shows the XRD of Sr-RG-C prepared by the combustion method. The diffraction pattern reveals the presence of crystalline RuO_2_ clusters as indicated by the appearance of typical diffraction peaks at 2*θ* = 28°, 35.2°, 39.96°, 45.09°, 59.4° and 59.6°, corresponding to crystal faces of {110}, {101}, {200}, {210}, {002} and {301} orientations, respectively. This result is in good agreement with the XRD reference card of tetragonal RuO_2_ (reference card number: 04-015-7002)^45^. Some remaining residuals of soluble SrCl_2_ and insoluble SrSO_4_ are also observed, which could not be removed under the present preparation conditions. The source of Cl^−^ and SO_4_^2−^ ions are Ru precursors and GO (SO_4_^2−^ remains as impurity in GO during its preparation), respectively.

It should be noted that the diffraction pattern of the as-prepared composite does not show any peaks related to the “originally used” graphene oxide (GO) (see Fig. (S1)). This indicates that the preparative approach is successful, leading to a complete transformation of GO to RGO. The XRD chart did not show any diffraction peak related to the predicted SrRuO_3_ perovskite structure. There are three possible scenarios: i) SrRuO_3_ is not formed; ii) instead, an amorphous SrRuO_3_ structure is formed; or iii) a highly-dispersed SrRuO_3_ in the nanometer or sub-nano size range is formed that is difficult to be distinguished in the XRD data.

Microwave irradiation is based on an efficient superheating of the material so that the product may acquire different properties and structure^46^. Unlike the case of Sr-RG-C, XRD of Sr-RG-M (prepared by the microwave irradiation) shows the formation of Ru nanoparticles instead of RuO_2_ (Fig. (1b)). The following diffractions are observed in the XRD chart of Sr-RG-M at 2*θ* = 38.34°, 42°, 43.96°, 58°, 69°, corresponding to XRD reference card (04-001-2957) of hexagonal Ru^47^. Some remaining traces of SrCl_2_ and SrSO_4_ are also observed, but with lower intensity compared to Sr-RG-C (prepared by the combustion method). This indicates that microwave irradiation minimizes the formation of impurities within the sample.

XRD is considered a bulk analysis tool with high depth profiling and is convenient to provide some information on the structure of the bulk and surface of a sample. However, taking into account the XRD lower detection limit it is challenging to extract quantitative information for subnano-meter size particles from any spectra. For our prepared samples, it is thus difficult to resolve the signal from the baseline noise. Thus, we believe that XRD can be reliable to elucidate the structure of Sr-RG-C, but additional surface analysis tools should be used for any further assessment.

XPS experiments are used to gain more information about the chemical composition, electronic state of different elements and their relative ratios in the prepared composites. Figure (1c–f) show the XPS spectra of Sr-RG-C and Sr-RG-M, allowing comparing the structures obtained by the different preparation routes described above. The C-1*s* spectra of both samples indicate the successful reduction of GO into RGO. The ratios between C–C/C=C (284.4 ± 1.0 eV) and oxygenated peaks (C=O; 287.8 ± 0.1 eV) are calculated as 5.9 and 1.2 for Sr-RG-C and Sr-RG-M, respectively. This indicates that higher reduction efficiency is realized when using the combustion method.

The peak at 279.9 ± 0.1 eV in both samples is assigned for Sr 3*p*_1/2_^48^. The Ru 3*d* peak in Sr-RG-C sample (prepared by combustion) is deconvoluted into four peak components at 280.9 ± 0.1 eV and 285.0 ± 0.1 eV that correspond to Ru 3*d*_5/2_ and Ru 3*d*_3/5_ of RuO_2,_ and at 282.6 ± 0.1 eV and 286.56 ± 0.1 eV that correspond to Ru 3*d*_5/2_ and Ru 3*d*_3/2_ in SrRuO_3_, respectively.

On the other hand, the Ru 3*d* peak in Sr-RG-M sample (prepared by microwave treatment) is deconvoluted into 5 peaks at 280.5 and 284.2 eV that correspond to Ru 3*d*_5/2_ and Ru 3*d*_3/2_ of metallic Ru, and at 281.9, 283.1 and 286.3 eV that correspond to Ru 3*d*_5/2_, its satellite and Ru 3*d*_3/2_ of SrRuO_3_.

The O 1*s* spectra of Sr-RG-C (Fig. 1e) is deconvoluted into three peak components, one at 528.6 eV is assigned to perovskitic oxygen^49^, the second at 529.7 eV is assigned to Ru-O* in RuO_2_^3^, and the peak at 531.4 ± 0.1 eV is assigned to graphenic C–O*/C=O* ^50^. In case of Sr-RG-M, the O 1*s* (Fig. 1f) peak could be deconvoluted to three peak components, one at 528.9 ± 0.1 eV assigned for the perovskitic oxygen, the second at 530.4 ± 0.1 eV corresponding to graphenic C–O* and/or adsorbed –OH^49^, the third component at 532.3 ± 0.1 eV assigned for graphenic C=O*.

The XPS data of Sr 3*d* in Sr-RG-C are shown in Supplementary Fig. (S2a). The main 3*d* peak is deconvoluted into four components: two peaks at 132.0 ± 0.1 eV and 133.8 ± 0.1 eV corresponding to Sr 3*d*_5/2_ and Sr 3*d*_3/2_ of Sr^2+^ in the perovskite structure with a binding energy separation of 1.8 eV, which is in agreement with the doublet splitting of Sr 3*d*. The other two peaks at 133.4 ± 0.1 eV and 135.1 ± 0.1 eV corresponding to Sr 3*d*_5/2_ and Sr 3*d*_3/2_ of SrCl_2_.

For the microwave prepared sample (Sr-RG-M), the Sr 3*d* spectrum is deconvoluted into four peaks at 132.8 and 134.6 ± 0.1 eV corresponding to Sr 3*d*_5/2_ and Sr 3*d*_3/2_ of Sr^2+^ in the perovskite structure and at 134.6 and 135.5 ± 0.1 eV corresponding to Sr 3*d*_5/2_ and Sr 3*d*_3/2_ of SrSO_4_.

A quantitative analysis of the atomic surface percentages/ratios of different elements in the prepared composites is also done based on the atomic sensitivity corrected (normalized) intensities for different lines (the results are summarized in Table 1)^51^. The ratio of Sr 3*d*:Ru 3*d*:O1*s* deconvoluted peaks are assigned for SrRuO_3_ to ascertain the *AB*O_3_ structure. Thus, the calculated Sr 3*d*: Ru 3*d*: O1*s*_p_ ratios in Sr-RG-C and Sr-RG-M are 1.2:1:3.1 and 1:1.3:3.1 that are consistent with *AB*O_3_ structure. The appearance of XPS components of SrRuO_3_ with high intensities indicates that SrRuO_3_ exists on the surface of both Sr-RG-C and Sr-RG-M composites.

**Table 1:** Atomic percentage of each element in *A*-RG nanocomposites deduced from XPS fitting.

Element	At% Sr-RG-C	At% Sr-RG-M	At% Ba-RG-C	At% Ba-RG-M	At% Ca-RG-C	At% Ca-RG-M
C	55.98	51.61	48.63	49.30	28.05	50.43
O	17.20	25.98	36.96	16.63	45.18	23.36
Ru	9.57	6.73	8.04	6.79	1.80	2.93
*A*-metal	6.56	4.34	2.03	3.15	4.57	2.02
Cl	10.69	11.33	4.33	17.24	1.66	9.00
N	—	—	—	6.89	18.75	12.23

**Figure 2:**
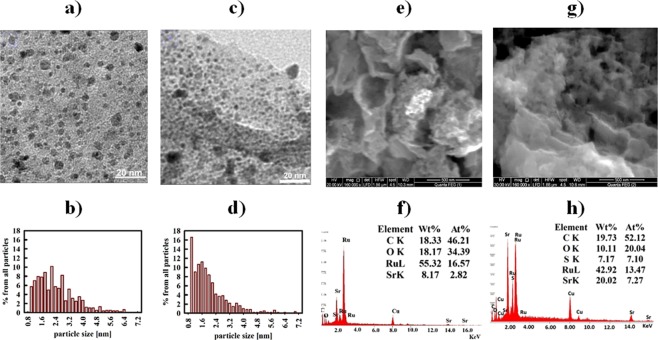
(**a**) TEM of Sr-RG-C, (**b**) particle size distribution of Sr-RG-C, (**c**) TEM of Sr-RG-M, (**d**) particle size distribution of Sr-RG-M, (**e**) SEM of Sr-RG-C, (**f**) the corresponding EDX analysis of Sr-RG-C, (**g**) SEM of Sr-RG-M and (**h**) the corresponding EDX analysis of Sr-RG-M.

The aforementioned findings indicate that SrRuO_3_ is prepared successfully using both methods. It is important to consider the results from the high-resolution electron microscopy to examine the dispersion of the perovskites on the surface. HR-TEM images of Sr-RG composites are depicted in Fig. (2a & c) showing typical wrinkled and highly-folded graphene sheets with homogeneously distributed nanoparticles grown over their surfaces. The particle size distribution shows that most of the particles in Sr-RG-C are in the range of 1–3 nm (see Fig. 2a,b). In case of Sr-RG-M, a significant fraction of particles is in the sub-nanometer size range of ≈0.9 nm in diameter (see Fig. 2c,d). The fraction of particles larger than 3.2 nm in both samples is relatively low which sufficiently explains the absence of SrRuO_3_ peaks in the XRD data and proves the successful preparation of sub-nano sized well-crystalline SrRuO_3_ in combination with RuO_2_ and Ru nanoparticles in case of Sr-RG-C and Sr-RG-M, respectively. The direct heating in air (as in case of combustion method) facilitates the oxidation of the fraction of metallic Ru in the sample to RuO_2_. The atomic ratio of SrRuO_3_ to RuO_2_ in Sr-RG-C as deduced from XPS measurements is nearly 2:3, while the ratio of SrRuO_3_ to Ru nanoparticles in Sr-RG-M is about 4:3.

The morphology of the formed material is strongly affected by the preparation method. Therefore, we investigated the effect of the preparation method on the morphology of the nanocomposites. Figure (2e–h) show the SEM images and the corresponding EDX analyses of Sr-RG-C and Sr-RG-M. Sr-RG-C in Fig. 2e shows fewer wrinkles and a highly porous structure, while Sr-RG-M in Fig. (2g) shows a sponge-like structure and the particles are distributed at the edges of graphene sheets in both cases. EDX analysis of Sr-RG-C shows that the atomic percentage of Ru is about 16.5 compared to 13.5 in the case of Sr-RG-M. A 7% sulfur content (as SrSO_4_) is observed in the microwave sample that is consistent with our obtained XRD results. Very fine particles are observed at the edges of the graphene sheets, being also consistent with the obtained TEM images.

### Ba-RG nanocomposites

**Figure 3:**
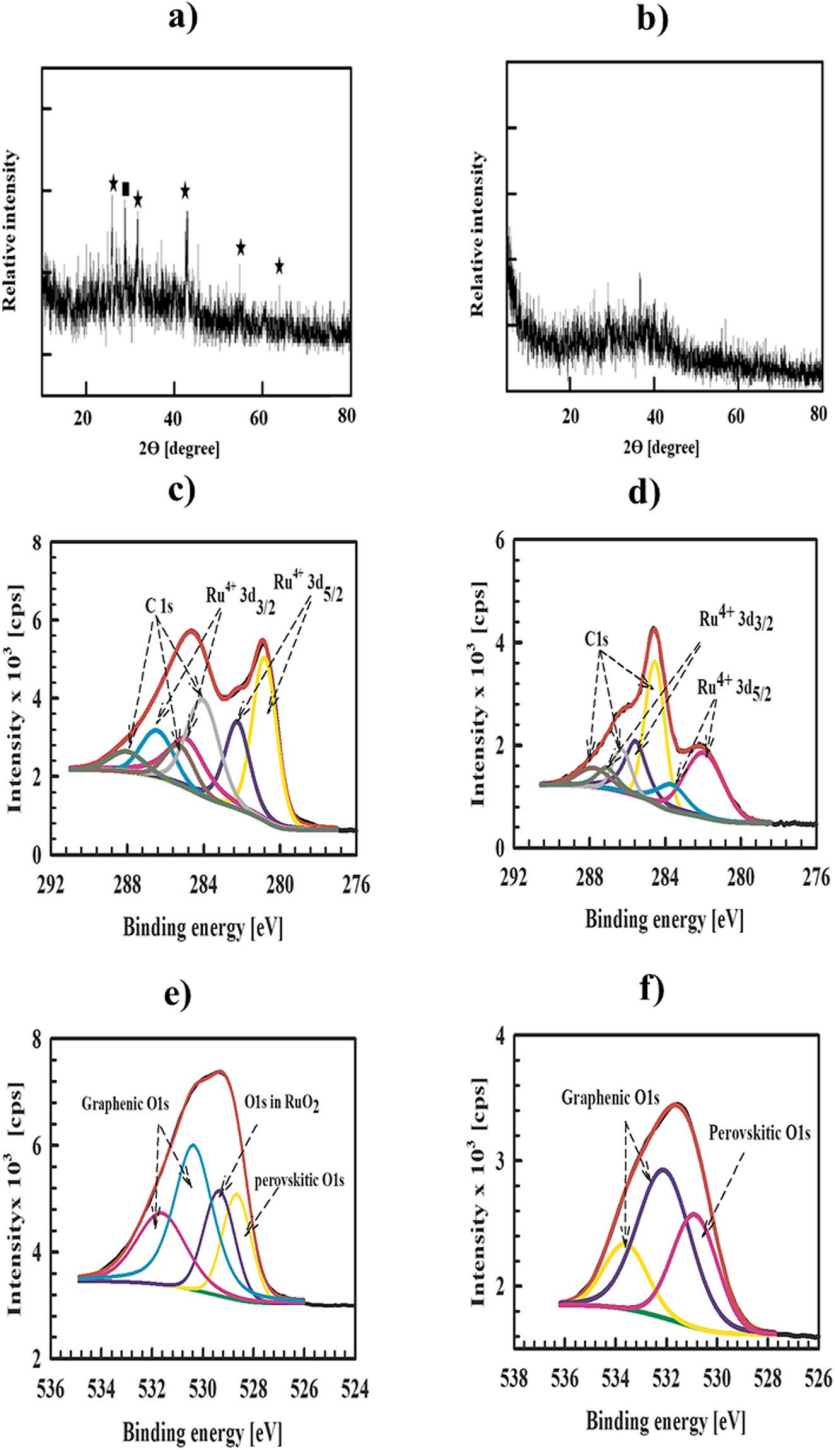
XRD of (**a**) Ba-RG-C, (**b**) Ba-RG-M (■ RuO_2_, and ★BaRuO_3_), (**c**) C 1*s* and Ru 3*d* of Ba-RG-C (**d**) C 1*s* and Ru 3*d* of Ba-RG-M, (**e**) O 1*s* of Ba-RG-C and (**f**) O 1*s* of Ba-RG-M.

The XRD spectrum of Ba-RG-C (Fig. 3a) shows peaks with low intensities at 2*θ* = 28°, being characteristic for RuO_2_. In addition, the diffraction peaks at 25.8°, 31.5°, 42.5°, 55° and 65° are characteristic for the {411}, {510}, {631}, {755} and {1020} facets of cubic BaRuO_3_ (reference card number 00-037-0846)^52^. As will be discussed later, the broad peaks recorded for Ba-RG-C indicate the formation of small-sized crystalline BaRuO_3_.

On the other hand, Ba-RG-M shows an amorphous structure without any distinct peaks that would be characteristic for a crystalline perovskite structure (see Fig. 3b). However, in order to really elucidate their structures again additional analysis techniques are necessary.

Figure (3c–f) show the XPS fitting of Ba-RG-C and Ba-RG-M. In both Ba-RG-C and Ba-RG-M C 1*s* is deconvoluted into three peak components, which are at 284.46, 285.2 and 288.0 eV for Ba-RG-C and at 284.5, 286.3 and 287.8 eV for Ba-RG-M, corresponding to graphenic carbon (C–C and/or C=C), C–O and C=O. The ratio between the C–C and the oxygenated peaks are 1.8 and 2 for Ba-RG-C and Ba-RG-M, respectively. This confirms the reduction of GO into RGO in both samples with a slightly more efficient reduction achieved by the microwave treatment. The Ru 3*d* levels in Ba-RG-C are deconvoluted into four peaks at: 280.9 and 284.89 eV corresponding to Ru 3*d*_5/2_ and Ru 3*d*_3/2_ of Ru^4+^ in RuO_2_ and at 282.2 and 286.4 eV corresponding to Ru 3*d*_5/2_ and Ru 3*d*_3/2_ of Ru^4+^ in the perovskitic structure. In the case of Ba-RG-M, Ru 3*d* is also deconvoluted into four components at 282, 283.6, 285.5, and 287.0 eV, corresponding to Ru 3*d*_5/2_ and Ru 3*d*_3/2_ of Ru^4+^ in the perovskitic structure and their satellites^53^. The O 1*s* spectra are very important for elucidating the structure of Ba-RG-C. As shown in Fig. (3e), O 1*s* of Ba-RG-C shows four deconvoluted peaks at 528.7 eV for perovskitic oxygen, at 529.4 eV for RuO_2_, as well as 530.4 and 531.6 eV for graphenic oxygens. In the case of Ba-RG-M, the components of O 1*s* are shifted to higher binding energies; 530.09, 532.09 and 533.6 eV. These may be due to the amorphous structure of BaRuO_3_ within the Ba-RG-M sample. The Ba 3*d* spectra of Ba-RG-C and Ba-RG-M (Fig. (S2c and d)) are deconvoluted into two peaks in both samples at 779.9 and 795.2 eV for Ba-RG-C and 780.35 and 795.69 eV for Ba-RG-M, corresponding to Ba 3*d*_5/2_ and Ba 3*d*_3/2_, respectively. Ba 3*d* in the case of Ba-RG-M is shifted to higher binding energies (about 0.5 eV). Consequently, we can conclude that crystalline BaRuO_3_ is formed by the combustion method, while the well-crystalline perovskite did not form in the case of Ba-RG-M and its amorphous structure is most likely due to the formation of an intermediate state of BaRuO_3_. Table 1 summarizes the calculated atomic percentage of each element in the different Ba-RG samples as deduced from our XPS analyses. In order to confirm the structure of BaRuO_3_, we calculated the ratio Ba 3*d*_5/2_: Ru 3*d*_5/2_: O 1*s* (perovskitic) for Ba-RG-C and Ba-RG-M. The obtained values are 1.1:1:3.2 and 1:1.3:3.2 for Ba-RG-C and Ba-RG-M, respectively, being in agreement with *AB*O_3_ perovskitic structures.

**Figure 4:**
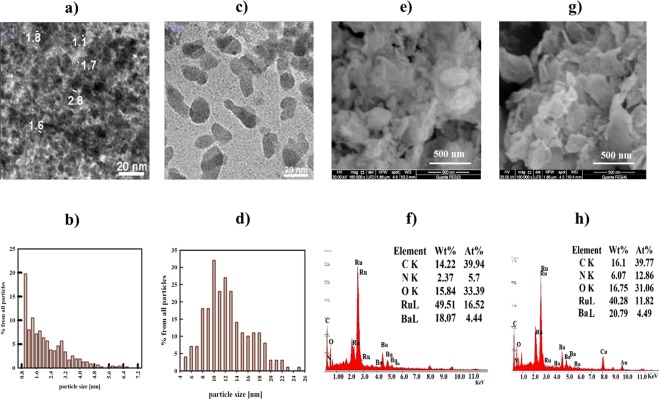
(**a**) TEM of Ba-RG-C, (**b**) particle size distribution of Ba-RG-C, (**c**) TEM of Ba-RG-M, (**d**) particle size distribution of Ba-RG-M, (**e**) SEM of Ba-RG-C, (**f**) the corresponding EDX analysis of Ba-RG-C, (**g**) SEM of Ba-RG-M and (**h**) the corresponding EDX analysis of Ba-RG-M.

HR-TEM images can provide extra information about the morphology of a sample. The corresponding images for both samples together with an analysis of the particle size distributions are shown in Fig. (4a–d). It can be noticed that Ba-RG-C shows less crimped graphene sheets and rather densely packed particles with less interspace separation in the distribution pattern. The particle size distribution shows that most of the particles are in the sub- to nanometer-range. This explains the large broadening of the XRD peaks of Ba-RG-C. On the other hand, HR-TEM of Ba-RG-M shows that the particles are larger and elongated, where the majority of particles are in the range of 5 to 25 nm with an average distribution of 10 nm. SAED-pattern of Ba-RG-C (see the inset of Fig. (S3c)) shows diffractions with *d*-spacing of: 1.58, 1.74, 1.86, 2.38 and 2.95 corresponding to {910}, {653}, {642}, {622}, {510} orientations, respectively, in the cubic BaRuO_3_ reference card. In contrast, no diffractions were observed for Ba-RG-M. This finding indicates an amorphous structure of BaRuO_3_ obtained by the microwave synthesis, while crystalline sub-nanometer sized BaRuO_3_ is formed via the combustion-based synthesis (*i.e*., Ba-RG-C).

The surface morphologies of our Ba-RG samples were also investigated by SEM (with EDX) as shown in Fig. (4e–h). Ba-RG-C (Fig. 4e) appears to have more wrinkles and lower porosity compared to Sr-RG-C, while the SEM of Ba-RG-M (Fig. 4g) reveals a fog-like shape with larger pore sizes compared to Ba-RG-C. Nitrogen is observed in the EDX of Ba-RG-C and Ba-RG-M that may be due to the formation of N-doped graphene. Ru atomic percentage in Ba-RG-C and Ba-RG-M is 16.5 and 11.8, respectively.

Generally, the morphology of composites prepared by the microwave method is more ordered and particle shapes are more identified compared to those prepared by the conventional combustion method. However, the conventional combustion method produces particles with relatively smaller sizes and higher Ru contents.

### Ca-RG nanocomposites

**Figure 5:**
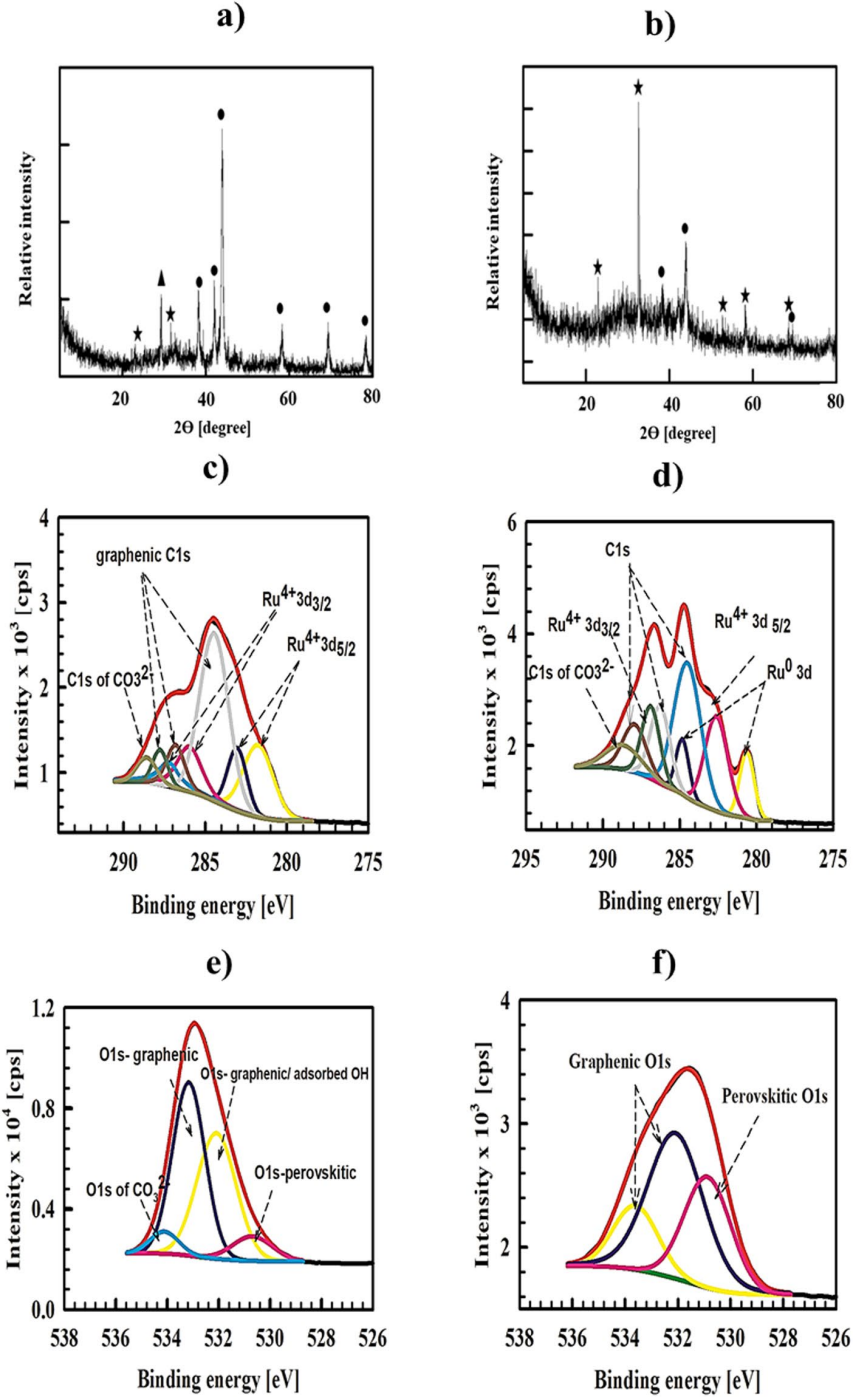
XRD of (**a**) Ca-RG-C, (**b**) Ca-RG-M (● metallic Ru, CaRuO_3_, and ▲ CaCO_3_), (**c**) C 1*s* and Ru 3*d* of Ca-RG-C (**d**) C 1*s* and Ru 3*d* of Ca-RG-M, (**e**) O 1*s* of Ca-RG-C and (**f**) O 1*s* of Ca-RG-M.

The XRD pattern of Ca-RG-C illustrated in Fig. (5a) reveals the dominant formation of Ru nanoparticles as the main phase together with a smaller fraction of a CaRuO_3_ phase with relative intensity of 6.7%. Surprisingly, the XRD of Ca-RG-M in Fig. (5b) reveals the formation of CaRuO_3_ as the main phase with “some” Ru nanoparticles as a secondary phase. The remaining insoluble CaCO_3_ appears in the XRD pattern of both samples.

The calculated *a*, *b* and *c* lattice parameters from *d*-spacings and the corresponding orientations are 5.52, 7.75 and 5.47 Å, respectively. These values compare well with the 5.53, 7.74 and 5.43 Å of CaRuO_3_ listed on the reference card (number: 04-015-2909)^54^. In this case, the diffractions of CaRuO_3_ appear with distinct intensities and can be identified from the XRD data. This indicates that the microwave irradiation combined with a highly exothermic redox reaction between Ca and Ru precursors and GO lead to the formation of highly-crystalline CaRuO_3_ without the need of any further calcination step.

This finding is supported by our XPS analysis as shown in Fig. (5c–f). The C 1*s* spectra in Ca-RG-C is also deconvoluted into four peaks at 284.4, 286.8, 287.8 and 288.5 eV, corresponding to graphenic C–C/C=C, C–O, C=O and carbonates, respectively. The same components also appear in the Ca-RG-M samples at 284.8, 286.27, 288 and 288.8 eV. The ratios between C–C/C=C to the oxygenated peaks are calculated as 3.5 and 1.33 for Ca-RG-C and Ca-RG-M.

The observed Ru 3*d* spectrum of Ca-RG-C does not show any component related to metallic Ru, although the XRD pattern shows metallic Ru to be the main phase in Ca-RG-C. Considering the sensitivity of Ru to air exposure, the absence of metallic Ru could be attributed to either the oxidation of the outermost metallic Ru shell or to the presence of CaRuO_3_ on the surface of the sample, suggesting the formation of Ru/CaRuO_3_ core/shell-nanoparticles.

The absence of the lattice O 1*s* of RuO_2_ in the O 1*s* spectra of Ca-RG-C makes the second suggestion more likely. Here, Ru 3*d* in Ca-RG-C is deconvoluted into four peaks at 281.7, 283.4, 285.9 and 287.3 eV, corresponding to Ru 3*d*_5/2_ and Ru 3*d*_3/2_ of Ru^4+^ and their satellites, respectively, in the perovskite structure^55^. On the other hand, the Ru 3*d* spectrum in Ca-RG-M is deconvoluted into four peaks at 280.5 and 284.7 eV corresponding to Ru 3*d*_5/2_ and Ru 3*d*_3/2_ of metallic Ru and at 283 and 287 eV corresponding to Ru 3*d*_5/2_ and Ru 3*d*_3/2_ of the perovskitic Ru^4+^. The O 1*s* spectrum is also deconvoluted into four peaks in both samples with binding energies at 530.7, 532.1, 533.2 and 534.2 eV for Ca-RG-C and at 531.0, 532.0, 532.8 and 534.3 eV for Ca-RG-M. These correspond to perovskitic lattice oxygen, graphenic C–O*, graphenic C=O* and oxygen in carbonate species. The higher binding energy for lattice oxygen in the case of CaRuO_3_ has also been reported in literature already^55^.

On the other hand, Ca 2*p* is deconvoluted into four peaks at 348.1 and 351.7 eV corresponding to Ca 2*p*_1/2_ and Ca 2*p*_3/2_ of Ca^2+^ in CaRuO_3_, and at 349.1 and 352.7 eV corresponding to Ca 2*p*_1/2_ and Ca 2*p*_3/2_ of CaCO_3_, while in the case of Ca-RG-M, the Ca 2*p* deconvoluted peaks are located at 347.8 and 351.3 eV correspond to Ca 2*p*_1/2_ and Ca 2*p*_3/2_, respectively of Ca^2+^ in CaRuO_3_ (see supplementary Fig. (S2e and f)).

It is worth mentioning that a N 1*s* peak is observed in most prepared samples with different percentages. The deconvolution of these N 1*s* signals shows the presence of N–C, N=C and N≡C bonds, indicating the preparation of N-doped graphene (Figure not shown). The atomic percentages of each element in Ca-RG-C and Ca-RG-M are also calculated for the quantitative analysis, the results are summarized in Table 1. The calculated Ca 2*p*_3/2_: Ru 3*d*_5/2_: O 1*s* (perovskitic) ratios are 1:1:3.2 and 1:1:2.9 for Ca-RG-C and Ca-RG-M, respectively.

**Figure 6:**
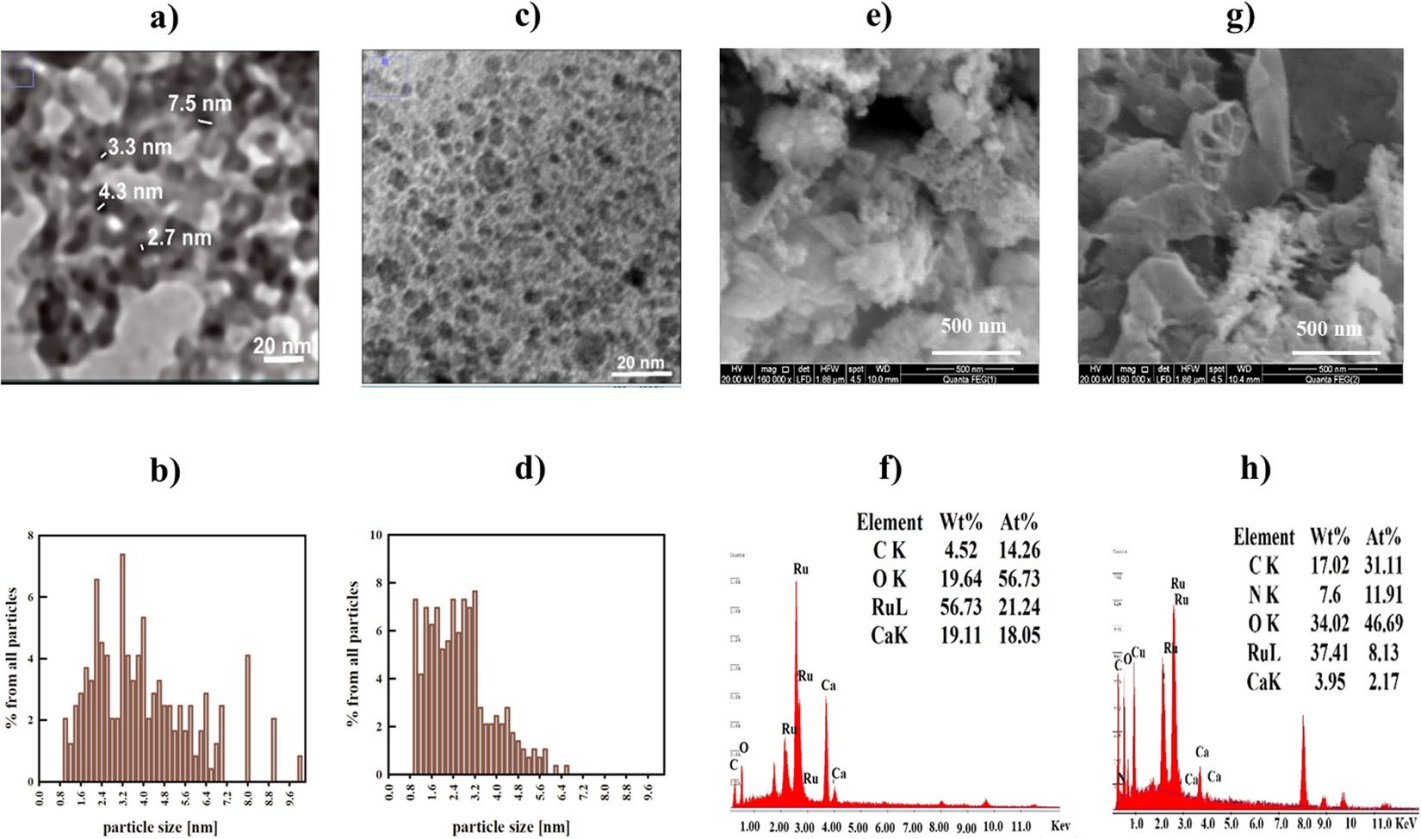
(**a**) TEM of Ca-RG-C, (**b**) particle size distribution of Ca-RG-C, (**c**) TEM of Ca-RG-M, (**d**) particle size distribution of Ca-RG-M, (**e**) SEM of Ca-RG-C, (**f**) the corresponding EDX analysis of Ca-RG-C, (**g**) SEM of Ca-RG-M and (**h**) the corresponding EDX analysis of Ca-RG-M.

The TEM image of Ca-RG-C (Fig. (6a)) shows graphene sheets with poorly distributed particles compared to Ca-RG-M, for which more compact structures are observable. Moreover, the microwave method results in graphene sheets with a high degree of folding, while the particles size distributions (Fig. 6b,d) show that both Ca-RG-C and Ca-RG-M have broad size distributions and larger particles (around 10 nm).

The broad particle size distribution and the presence of relatively larger particles explain the appearance of the diffractions of CaRuO_3_ in the XRD chart of Ca-RG composites compared to the other composites that have smaller sized particles with narrow size distributions. Our SEM images of Ca-RG-C and Ca-RG-M (Fig. 6e,g) reveal a morphology being consistent with what could be deduced from our TEM images. SEM of Ca-RG-C (Fig. (6e)) shows larger pores compared to other samples and fine particles appear on the graphene sheets. On the other hand, Ca-RG-M in Fig. (6g) shows a flower-like shape and has the most compact structure of all studied composites. The particles are also imbedded between the edges of graphene sheets. The high oxygen percentage observed in the EDX analyses is due to the hygroscopic nature of Ca salts that absorb water molecules from the atmosphere. A higher Ru content (21%) was observed in Ca-RG-C compared to 8.1% in the case of Ca-RG-M that represents the lowest Ru content in this sample class.

It should be mentioned that this is the first time CaRuO_3_ could be prepared with high crystallinity without a calcination step that usually requires temperatures as high as 650 °C)^55–57^.

### Ru/RGO nanocomposites in the absence of metal “*A*”

The XRD pattern of RG-C prepared by the same conditions and in absence of metal *A* salts are shown in supplementary Fig. (S4a). It appears that some Ru^3+^ ions are transformed into RuO_2_ as indicated by the appearance of low intensity diffractions at 2*θ* = 27.88°, 34.9°, 40° and 54° that are consistent with tetragonal RuO_2_^45^. The poor crystallinity may be an indication for unreacted Ru^3+^ ions. Upon dispersion of RG-C in DMF the solution assumes a yellowish color, indicating the presence of unreacted RuCl_3_.

The C 1*s*, O 1*s* and Ru 3*d* spectra of RG-C are shown in Fig. (S4b). The Ru 3*d* level is deconvoluted into four peaks: two peaks at 280.7 and 284.9 eV that correspond to Ru 3*d*_5/2_ and Ru 3*d*_3/2_ of RuO_2_ and additional two peaks with higher areas at 282.0 and 286.0 eV that correspond to Ru 3*d*_5/2_ and Ru 3*d*_3/2_ of RuCl_3_. This finding also proves that most Ru^3+^ ions are not completely converted into RuO_2_; they rather remain in the form of RuCl_3_. The XPS spectrum of O 1*s* (Fig. (S4c)) is deconvoluted into three peaks at 529.3, 530.7 and 532.7 eV corresponding to oxygen bonded to transition metal (RuO_2_), OH/C–O and O–C=O, respectively. The C 1*s* spectra are deconvoluted into three peaks at 284.4, 285.9 and 288.2 eV, corresponding to C–C/C=C, C–O and C=O, respectively. The total intensity of the C–C/C=C peak is higher than that of the oxygenated peaks, indicating the reduction of GO into RGO.

It is worth noting that in our previous work^58^ Ru-based RGO nanocomposites have been successfully prepared by the microwave method without using reducing or hazardous materials. We have found that a mixture of Ru and RuO_2_ nanoparticles is successfully loaded on RGO by adjusting the pH of the precursors to 8.0, while pure Ru nanoparticles are loaded on RGO sheets if the pH is adjusted to 4.0^58^.

**Figure 7:**
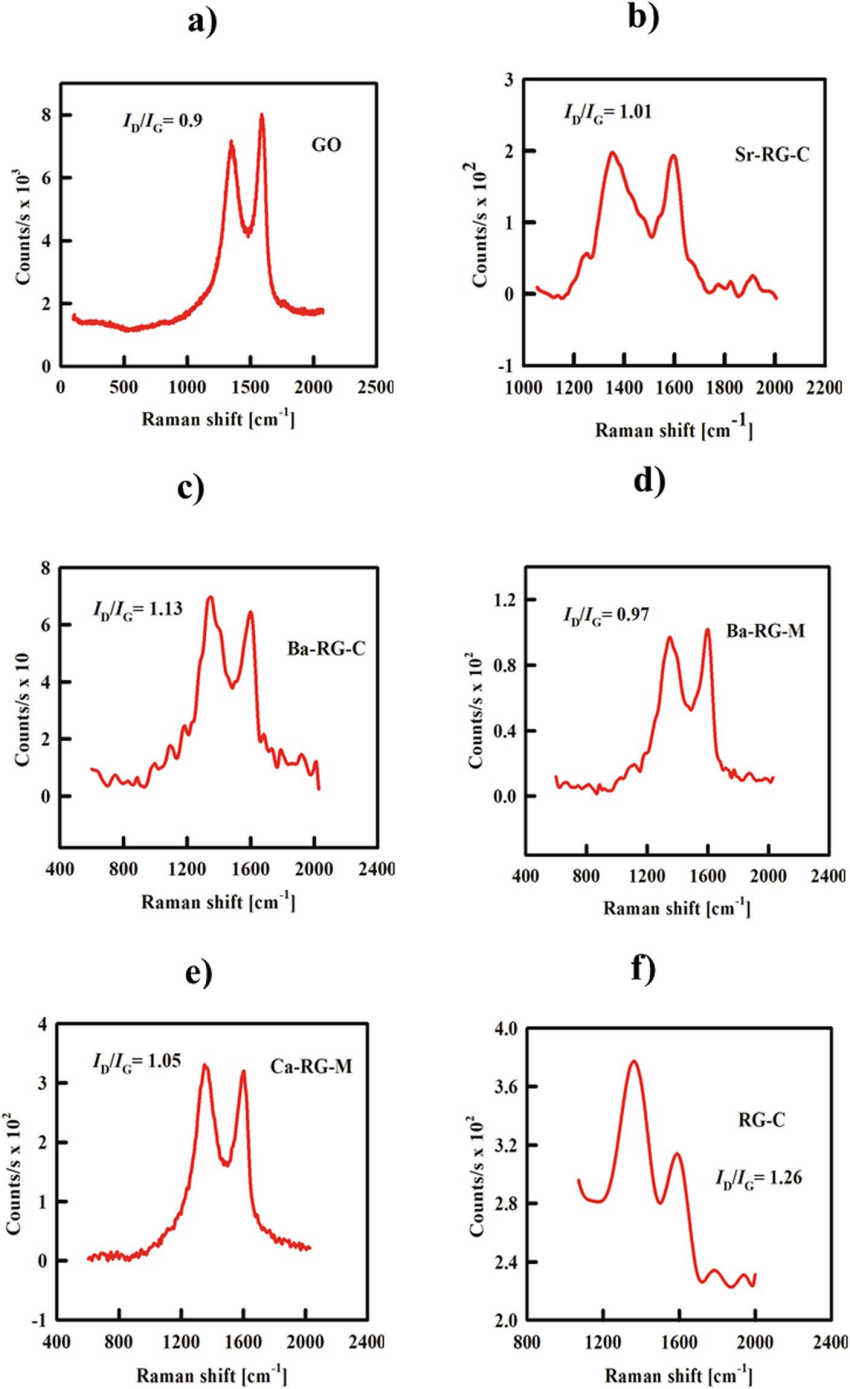
Raman spectra of (**a**) GO, (**b**) Sr-RG-C, (**c**) Ba-RG-C, (**d**) Ba-RG-M, (**e**) Ca-RG-M and (**f**) RG-C showing the region of *G* and *D* bands of GO and RGO to prove the successful reduction of GO into RGO.

Raman spectroscopy was used as additional evidence for the successful reduction of GO into RGO. Here, two bands appear for the GO sample: one at 1600 cm^−1^ (G-band) corresponding to the first scattering of the *E*_2g_ phonon of *sp*^2^ carbon and a second band at 1334.5 cm^−1^ (D-band) that arises from a breathing mode of *K*-point phonons of *A*_1g_ symmetry with an *I*_D_/*I*_G_ ratio 0.9. Another indication of the successful reduction of GO into RGO lies in the increased *I*_D_/*I*_G_ ratio. The shifts in wave number of both *G*- and *D*-bands are also indications for RGO formation. The Raman spectra of RG-C and *A*-RG nanocomposites prepared by the current method are shown in Fig. (7). An increase in the *I*_D_/*I*_G_ ratio is observed for all composites compared to GO.

## Conclusion

Nano-sized Ru-based perovskites/RGO nanocomposites were prepared using a “green”, one-pot low-temperature method. The preparation method is based on the redox reaction between a salt *A* (*A* = Ca, Ba or Sr), RuCl_3_ as well as GO, initiated by either conventional combustion or microwave irradiation. The structural analyses of the resulting composites revealed that sub-nano sized SrRuO_3_ was formed in combination with RuO_2_ or Ru nanoparticles when using the conventional combustion or microwave irradiation, respectively. For the first time, highly crystalline nano-sized CaRuO_3_ is formed as the main phase when using the microwave method. For BaRuO_3_ particles with a diameter of ~1 nm was successfully prepared together with RuO_2_ using the conventional combustion route, while amorphous BaRuO_3_ was prepared by microwave irradiation.

No further calcination step was needed to prepare such composites. Generally, the microwave irradiation route leads to ordered and well-distinct nanoparticles compared to those prepared by the conventional combustion method, which are usually smaller in size with highly porous structures. Consequently, microwave irradiation seems to promote the preparation of Ru nanoparticles, while the direct hotplate heating (conventional combustion method) promotes RuO_2_ formation.

## Supplementary information

Supplement Figures
