# Supplementary material for: Synthesis, structural and morphological characterizations of nano-Ru-based perovskites/RGO composites
Source: Sci Rep. 2019 May 28;9:7948. doi: 10.1038/s41598-019-43726-1 (PMC6538664; doi:10.1038/s41598-019-43726-1)
Supplement: Supplementary file 1 — Supplement Figures [file 41598_2019_43726_MOESM1_ESM.pdf]

Synthesis, structural and morphological characterizations of nano-Ru-based perovskites/RGO composites

Ahmed Galal,[*a] Hagar K. Hassan,[a] Nada F. Atta,[a] Ali M. Abdel-Mageed,[ab] Timo Jacob,[cde]
[a]Department of Chemistry, Faculty of Science, Cairo University, 12613 Giza, Egypt
E-mail: galal@sci.cu.edu.eg
[b]Institute of Surface Chemistry and Catalysis, Ulm University, 89069 Ulm, Germany
[c]Institute of Electrochemistry, Ulm University, 89081 Ulm, Germany
[d]Helmholtz-Institute-Ulm (HIU) [d] Helmholtzstr. 11, 89081 Ulm, Germany
[e]Karlsruhe Institute of Technology (KIT), P.O. Box 3640, 76021 Karlsruhe, Germany

Supplementary Figures

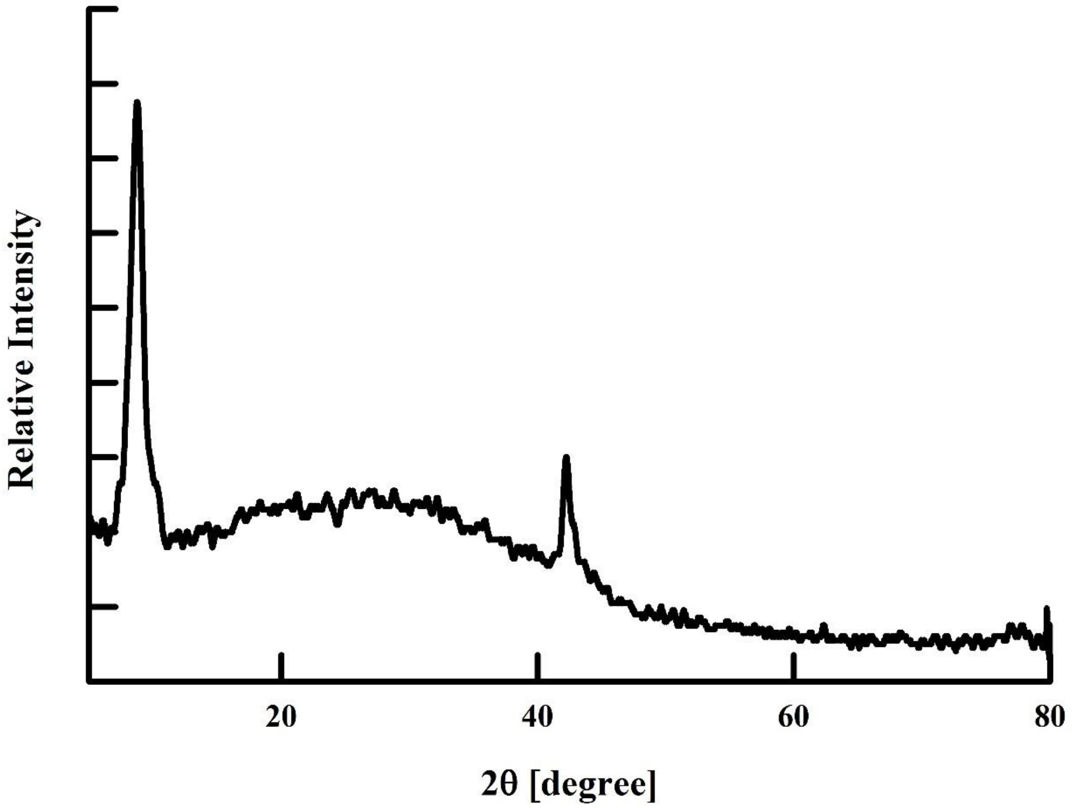

S1: XRD of graphene oxide.

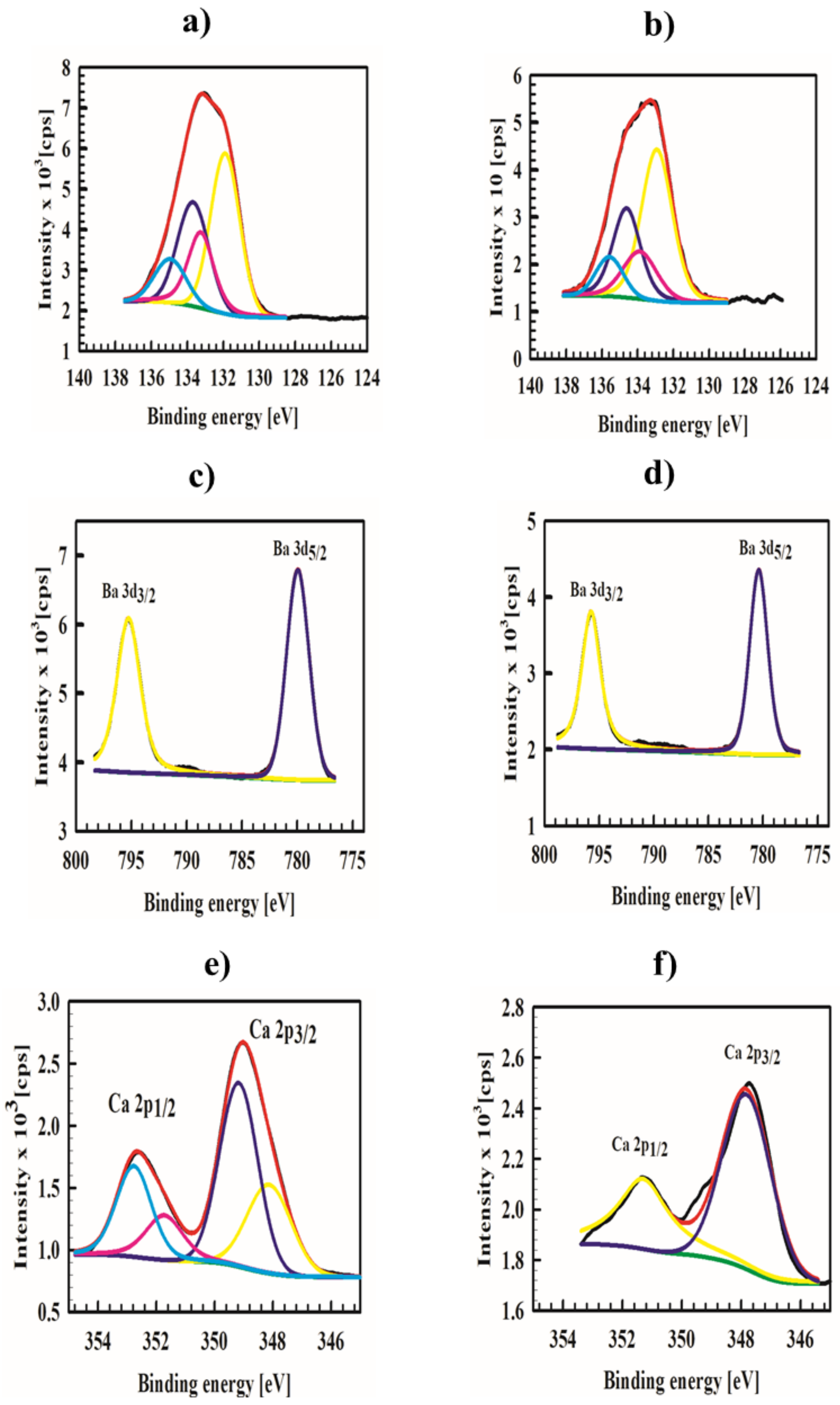

S2: Sr 3*d* spectrum of a) Sr-RG-C and b) Sr-RG-M, Ba 3*d* spectra of c) Ba-RG-C and d) Ba-RG-M and Ca 2*p* Spectra of e) Ca-RG-C and f) Ca-RG-M.

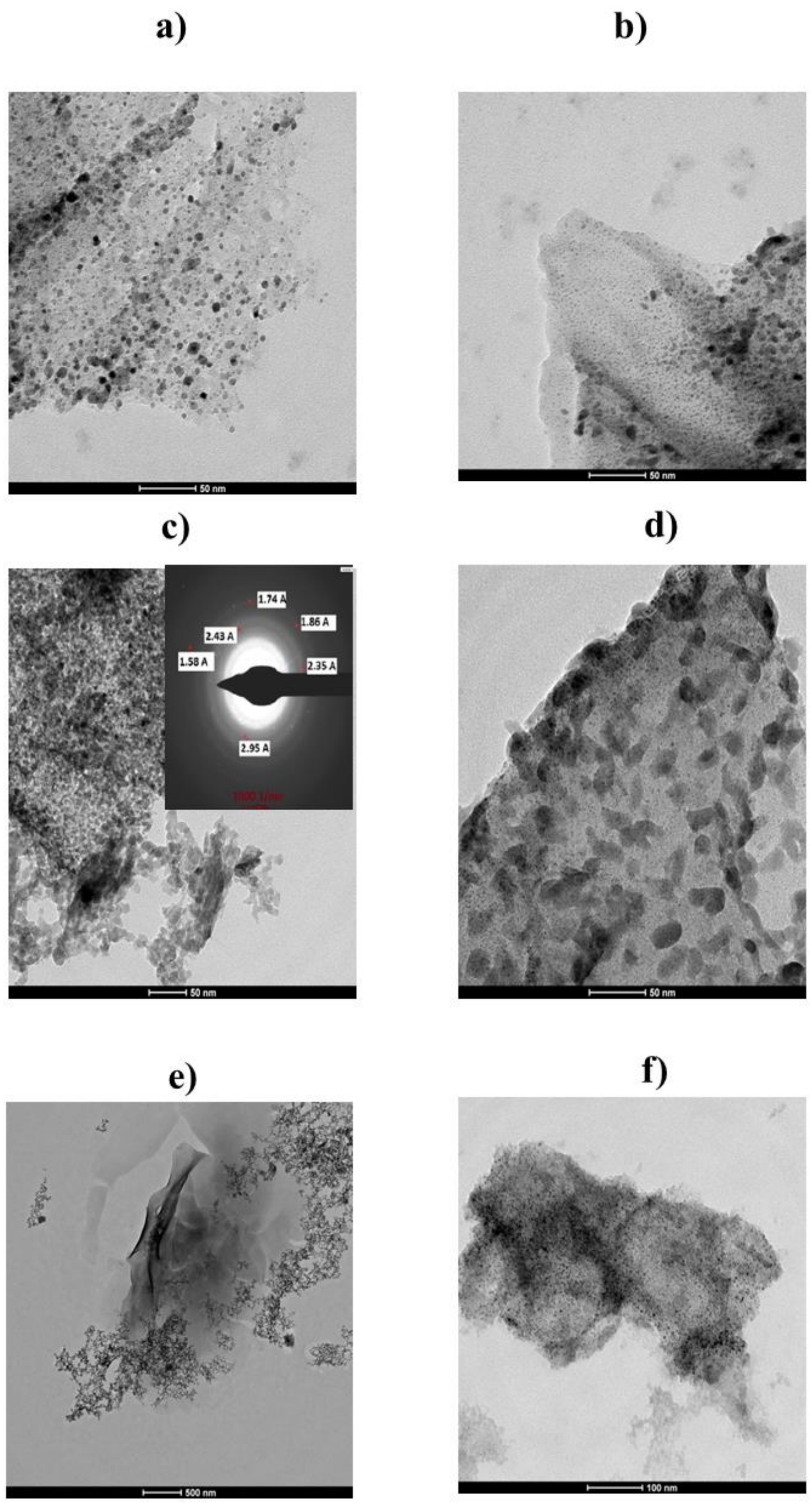

S3: Large-scale TEM images of *A*-RG nanocomposites showing graphene folding and particle distributions a) Sr-RG-C, b) Sr-RG-M, c) Ba-RG-C (inset: the corresponding SAED pattern), d) Ba-RG-M, e) Ca-RG-C and f) Ca-RG-M.

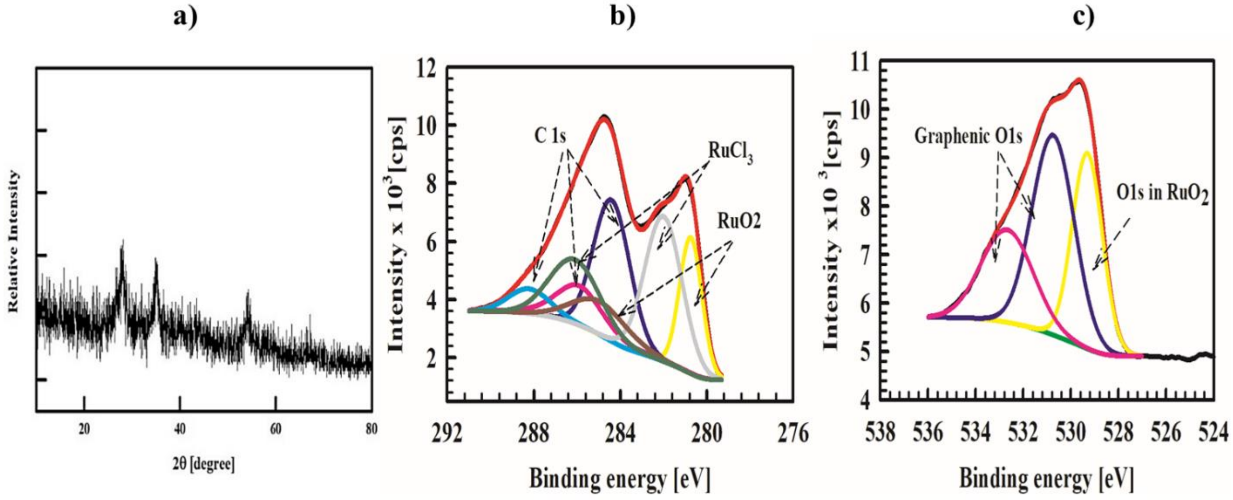

S4: a) XRD of RG-C, b) C 1*s* and Ru 3*d* spectra of RG-C and c) O 1*s* spectrum of RG-C.
